# Analyses of Antioxidative Response in Tomato (*Solanum lycopersicum* L.) Grown with Biochar and PGPMs

**DOI:** 10.3390/antiox14121482

**Published:** 2025-12-10

**Authors:** Silvia Carlo, Marta Trazza, Luca Pagano, Marta Marmiroli

**Affiliations:** 1 Department of Chemistry, Life Sciences and Environmental Sustainability, University of Parma, 43124 Parma, Italy; silvia.carlo@unipr.it (S.C.); marta.trazza@unipr.it (M.T.); 2 Consorzio Interuniversitario Nazionale per le Scienze Ambientali (CINSA), University of Parma, 43124 Parma, Italy; luca.pagano@unipr.it

**Keywords:** oxidative stress, antioxidants, plant response, soil health

## Abstract

*Solanum lycopersicum* plants were grown in pots amended with biochar and PGPMs (plant growth-promoting microorganisms: *Pseudomonas fluorescens* and *Azotobacter chroococcum*), applied singularly and in combination, for three months, after which plants and soils were collected, divided into treatment groups based on organs, and analyzed. The following biochemical markers were studied: cellular respiration, shoot fresh and dry weight, root fresh weight, photosynthetic pigments (chlorophyll a, chlorophyll b, and carotenoids), membrane lipid peroxidation, proline content, total antioxidant capacity (DPPH and ABTS assay), hydrogen peroxide, ascorbic acid, total phenolic content, enzymatic activity (SOD, POD, CAT, and APX), total soluble sugar content, and total protein content. Also, soil parameters, such as pH, EC, total enzymatic activity, active carbon, and respiration, were measured. While biochar alone induced root H_2_O_2_ accumulation, its co-application with PGPMs turned this signal into a systemic trigger for defense, enhancing the antioxidant capacity and the production of proline, phenolics, and ascorbic acid without causing oxidative damage. At the soil level, microorganisms counteracted biochar’s inhibitory effects on enzymatic activity and intensified labile carbon use, indicating a more dynamic rhizosphere. Multivariate analysis confirmed that the combined treatment remodulated the plant–soil system, converting a stress factor into a resilience enhancer. This synergy underscores the role of biochar as an effective microbial carrier and PGPM consortia as bioactivators, together providing a powerful tool to prime crops against climate stress while preserving soil health.

## 1. Introduction

Technological advances in agriculture, including the Green Revolution and recent digital innovations, have substantially increased crop yields over the past decades; however, these gains have also come at considerable environmental and social costs, notably through heightened agrochemical use and reduced dietary diversity [1]. Climate variability and projected climate change, including shifts in temperature, precipitation patterns, and the frequency of extreme weather events, are expected to place additional pressure on future crop production and food systems [2]. Furthermore, food systems are facing unprecedented challenges as they attempt simultaneously to accommodate a growing global population and to adapt to the impacts of climate change [1]. In this context, vegetable production is particularly critical, given its importance in balanced diets and its inherently resource-intensive cultivation [3]. However, vegetable crops are highly vulnerable to a wide range of biotic and abiotic stresses, including nutrient deficiencies, which induce genetic, metabolic, physiological, and phenotypic changes in plants, ultimately leading to reduced yield and quality [4]. Moreover, such physiological disruptions have been shown to reduce yield and quality in several vegetable crops, including tomatoes, beans, and leafy greens [5]. Tomato (*Solanum lycopersicum* L.) is a major dietary source of essential macro- and micronutrients. The fruit is especially rich in potassium (K), the most abundant mineral, contributing up to 7.7% of the daily reference intake (DRI) per 100 g serving, depending on the cultivar. Other important minerals include magnesium (Mg), phosphorus (P), calcium (Ca), iron (Fe), and zinc (Zn), although their concentrations can vary according to genotype and cultivation conditions [6,7]. Tomatoes also contain appreciable levels of vitamins (in particular vitamin C), dietary fiber, and bioactive compounds such as carotenoids (e.g., lycopene) and phenolics, which possess recognized antioxidant properties. Nutrient density and composition are influenced by both genetic factors and environmental conditions, with greenhouse-grown tomatoes often exhibiting higher mineral contents than their field-grown counterparts [8,9].

Tomato is, however, a highly water-demanding crop, typically requiring about 500–600 mm of water per growing season, and is sensitive to variability in temperature and rainfall [10]. Its growing season is frequently affected by scarce or excessive precipitation and by extreme temperatures above 40 °C, leading to recurrent water and heat stress during critical phenological stages; such conditions disrupt vegetative and reproductive processes and result in reduced productivity [11]. Between 2023 and 2025, tomato cultivation has shown both global expansion and marked regional volatility. Global production for 2025 is forecast to increase modestly, with an estimated rise of 4.1% over this period, primarily driven by growth in China, India, and Turkey, which are consolidating their dominance in the sector. By contrast, the United States and the European Union are expected to experience a slight decline in total output, largely attributable to water scarcity and heat stress in major producing regions such as California, and to contractions in traditional growing areas such as Spain and Italy, where shortened crop seasons and an increased frequency of extreme weather events are projected to reduce yields and fruit quality [12,13].

Agronomic strategies such as the use of plant growth-promoting microorganisms (PGPM) and novel amendments, including biochar, are increasingly recognized as effective means to mitigate the negative impacts of climate change on crop production [14,15]. Recent research highlights biochar as a promising amendment to enhance soil quality and support sustainable agriculture. Key indicators of good soil quality include favorable texture, nutrient and moisture retention, and the capacity to support robust microbial activity [16]. Evidence suggests that biochar improves soil health and crop yields while preserving overall soil integrity [17]. Achieving higher crop yields without reliance on synthetic fertilizers or additives remains a central challenge in sustainable agriculture [18], particularly as inorganic fertilizer use has previously driven productivity increases at the expense of long-term soil fertility [19]. Biofertilizers, including PGPMs, are eco-friendly alternatives that underpin sustainable agronomic practices. These products contain living microorganisms that enhance soil nutrient status through organic matter decomposition, thereby facilitating mineral nutrient uptake by plants and contributing to improved crop productivity over the longer term [20,21,22]. Bacterial biofertilizers are pivotal in fixing nitrogen, solubilizing phosphorus, potassium, zinc, and silica, thus aiding the availability of both macro- and micronutrients in the soil [5,23,24]. In addition, biofertilizers promote plant growth by enhancing soil fertility, releasing growth hormones, producing antibiotics, and biodegrading organic matter [5,25]. Scientific literature increasingly indicates that both biochar and plant growth-promoting microbes can enhance soil fertility [26], while current research demonstrates that their combined use significantly modulates soil dynamics and improves productivity [27]. This study aimed to assess the impact of biochar and PGPM application, individually and in combination, on the oxidative status of tomato plants cultivated in soil under controlled greenhouse conditions. Biochar was sourced from plant residues, and a consortium of PGPMs was assembled. The PGPM strains *Pseudomonas fluorescens* DR54 and *Azotobacter chroococcum* LS-132 were selected for their well-characterized nitrogen-fixing and phosphorus-solubilizing capacities, as well as their previously demonstrated rhizosphere-colonization ability. Their combined use has been validated by Tabacchioni et al. [28] as an effective biostimulant consortium capable of enhancing nutrient cycling and modulating plant redox responses. This study provides a novel integrated plant–soil multivariate perspective on how biochar and PGPMs interact to modulate redox signaling. Unlike prior studies, this one focused primarily on growth or nutrient uptake. Our work evaluated the priming-like redox response based on H_2_O_2_ accumulation, antioxidant capacity, and soil biochemical activity, considering the overall plant-soil system.

## 2. Materials and Methods

All reagents used in the experiments were purchased from Merck (Darmstadt, Germany) unless specified otherwise. All spectrophotometric analyses were performed with the spectrophotometer Varian Cary 50 (Thermo Fisher, Waltham, MA, USA) and the microplate reader iMark Microplate Absorbance Reader (Bio-Rad Laboratories, Hercules, CA, USA).

### 2.1. Bacterial Growth and Biochar Functionalization

Two strains of PGPMs (*Pseudomonas fluorescens* DR54 and *Azotobacter chroococcum* LS132) [28] were used in this study, grown in LB medium (tryptone 10 g/L, NaCl 10 g/L, yeast extract 5 g/L) and incubated overnight at 28 °C in a thermostatic orbital shaker (Heidolph unimax 2010, Fisher, Segrate, Italy) set at 200 rpm. After 24 h, cultures were diluted (1:10 ratio), and optical density at 600 nm (OD_600_) was measured spectrophotometrically to estimate the corresponding colony-forming units per milliliter (CFU/mL). For each strain, an aliquot corresponding to 10^8^ CFU/mL was prepared. The two bacterial suspensions were then combined in a 1:1 ratio, resuspended in fresh LB medium, and used for root inoculation and biochar functionalization. Biochar derived from various plant feedstocks, including sunflower residues, was employed as a soil amendment. Detailed biochar characterization is provided in Table S1 and Figure S1. Biochar particles had an average size of 5 mm, obtained by sieving prior to functionalization. Functionalization was carried out under sterile conditions by adsorbing 1 mL of the bacterial suspension (10^8^ CFU/mL) per gram of biochar. The biochar was then incubated at room temperature for 24 h to facilitate bacterial adhesion prior to soil application.

### 2.2. Plant Treatment and Sample Collection

*Solanum lycopersicum* L. (cv. Ciliegino; BLUMEN Group, Piacenza, Italy) seeds were germinated in standard potting soil (Compo GmbH, Münster, Germany) in seedbeds. Seedlings were maintained in a growth chamber (24 °C, 16 h light/8 h dark photoperiod) until the emergence of the first two fully expanded leaves. After three weeks, the plants were transplanted into pots (25 cm diameter × 25 cm height) containing 3 kg of soil and irrigated with 300 mL of tap water twice a week, with three biological replicates per treatment group. Four experimental conditions were established: (1) untreated control (nt); (2) soil amended with 1% (*w*/*w*) biochar (char); (3) plants inoculated at the root level with a bacterial suspension (10^8^ CFU/mL) (PGPM), using the same total bacterial load as that applied during biochar functionalization (30 × 10^8^ CFU per plant); and (4) soil amended with 1% (*w*/*w*) biochar functionalized with the bacterial suspension (comb). Plants were grown in a greenhouse under controlled conditions (24 ± 2 °C, relative humidity 55–65%, 16 h light/8 h dark photoperiod) for a period of three months before sample collection. No other stress, biotic or abiotic, was applied to plants at the time of the experiment. Soil samples were air-dried for subsequent analysis. Leaves and roots were thoroughly washed, then a part was air dried in an oven at 70 °C for 24 h, and a part was immediately flash-frozen in liquid nitrogen and stored at −80 °C until use for physiological and biochemical assays.

### 2.3. Soil Analysis

Physicochemical analyses of the soil were performed following sieving through a 2 mm mesh.

#### 2.3.1. Determination of Soil pH and Electrical Conductivity (EC)

Soil pH (EPA Method 9045D) and electrical conductivity (EC) (FAO Standard Operating Procedure (SOP) on soil electrical conductivity) were assessed according to the standardized protocols with minor modifications. Specifically, 1 g of soil from each sample was suspended in 20 mL of double-distilled water (ddH_2_O) and shaken continuously for two hours. After incubation, pH was measured using a pH meter (Seven Compact Duo, Mettler Toledo, Columbus, OH, USA). For EC determination, the suspension was filtered through Whatman grade 1 filter paper, and EC was measured using a conductivity meter (Seven Compact Duo, Mettler Toledo, Columbus, OH, USA).

#### 2.3.2. Active Carbon (POXC) and Soil Respiration

Active carbon (Permanganate-Oxidizable Carbon, POXC) and soil respiration were measured according to Standard Operating Procedures (SOP) CSH 04 and CSH 06, respectively [29], with minor modifications. For POXC determination, 0.25 g of dried soil was suspended in 20 mL of a 0.02 M potassium permanganate (KMnO_4_, pH 7.2) solution and shaken for 2 min. The mixture was then allowed to stand for an additional 8 min to enable the oxidation of the active carbon in the soil. The reaction was stopped by diluting the supernatants in ddH_2_O (1:100), and the absorbance of the resulting solution was measured at 550 nm to assess the decrease in the characteristic purple color of the permanganate solution. Absorbance values were interpolated against a standard calibration curve prepared from KMnO_4_ solutions of known concentrations (0.005 M, 0.01 M, and 0.02 M). The concentration of active carbon in the soil was then calculated following the methodology outlined in SOP CSH 04 [29]. Soil respiration rate was assessed by quantifying the carbon dioxide (CO_2_) released as an indicator of microbial metabolic activity, using the sealed chamber alkali trap respirometry method: 2 g of air-dried soil was rehydrated and placed in a sealed jar containing a cuvette with 0.9 mL of 0.5 M KOH solution. After a 4-day incubation period, the amount of CO_2_ released was estimated by comparing the change in EC of the KOH solution incubated with soil to that of a blank (KOH incubated without soil) as reported in SOP CSH 06 [29].

#### 2.3.3. Total Soil Enzymatic Activity

Microbial enzymatic activity was assessed using the fluorescein diacetate (FDA) hydrolysis assay [30]. Following the preparation step described in ISO 11063:2020 [31] for soil microbial analyses, 2 g of soil was incubated overnight in 20 mL of 60 mM phosphate buffer. Then, 200 µL of FDA solution (1 mg/mL) was added to the suspension and shaken for 1 h at room temperature in the dark. After allowing the mixture to settle, the supernatant was collected and centrifuged to remove soil particles. The absorbance of the clarified solution was then measured at 490 nm.

### 2.4. Morphological, Physiological, and Biochemical Analysis in Plants

Following plant harvest, the fresh weight of shoots and roots, as well as the dry weight of shoots, was recorded.

#### 2.4.1. Cellular Respiration and Lipid Peroxidation

Cellular respiration in fresh leaves was evaluated using the 2,3,5-triphenyltetrazolium chloride (TTC) assay, while lipid peroxidation in roots and leaves was assessed via the thiobarbituric acid reactive substance (TBAR) assay, according to Pagano et al. [32], with minor modifications. Specifically, a 15 mm diameter leaf disc was excised using a cork borer and incubated in 4 mL of TTC buffer (TTC 0.18 M, 78% Na_2_HPO_4_·H_2_O 0.05 M, 22% KH_2_PO_4_ 0.05 M) for 15 h at 30 °C, in the dark. After incubation, the discs were rinsed twice with ddH_2_O, and the resulting formazan was extracted in 5 mL of 95% ethanol at 80 °C for 10 min. Absorbance of the extract was then measured at 530 nm. Lipid peroxidation of cellular membranes was assessed by quantifying malondialdehyde (MDA) content in flash-frozen leaf and root tissues. Approximately 100 mg of tissue was ground in liquid nitrogen using a mortar and pestle and then homogenized in 0.1% (*w*/*v*) trichloroacetic acid (TCA). The homogenate was centrifuged at 12,000× *g* for 15 min at 4 °C. An aliquot of 500 µL of the resulting supernatant was mixed with 1 mL of reaction solution containing 0.5% TBA dissolved in 20% TCA. The mixture was incubated for 30 min at 95 °C to allow the formation of the TBA–MDA complex. The reaction was stopped by rapid cooling on ice, and absorbance was measured at 532 nm. MDA concentration was determined by interpolation from a standard calibration curve (0–50 µM).

#### 2.4.2. Photosynthetic Activity

Photosynthetic pigments were extracted by incubating 50 mg of powdered leaf tissue with 500 µL of cold 95% (*v*/*v*) acetone on ice for 10 min. The homogenate was then centrifuged to separate plant debris from the pigment-containing acetone extract [33]. Chlorophyll a (Chl a), chlorophyll b (Chl b), and carotenoid contents were quantified spectrophotometrically by measuring absorbance at 662 nm, 647 nm, and 480 nm, respectively, and calculated as reported by Wellburn [34]. Pigment concentrations are expressed as micrograms per gram of fresh leaf weight (µg/g FW).

#### 2.4.3. Proline Content in Leaves

Proline levels were determined in 100 mg of flash-frozen powdered leaf tissue homogenized in 1 mL of 3% (*w*/*v*) sulphosalicylic acid [35]. After centrifugation (10 min, 14,000× *g*, 4 °C), the supernatant was collected and kept on ice. For the colorimetric assay, 50 µL of extract was mixed with 1 mL of reaction solution comprising 3% (*w*/*v*) sulphosalicylic acid, glacial acetic acid, and 2.5% (*w*/*v*) ninhydrin in a 1:1:2 ratio. The mixture was incubated at 100 °C for 15 min, then rapidly cooled on ice. Absorbance was measured at 520 nm. Proline concentrations were calculated via interpolation against a standard curve (0–30 µg/mL) and expressed as µg/g of fresh weight (FW).

#### 2.4.4. Hydrogen Peroxide Content

Hydrogen peroxide (H_2_O_2_) levels in root and leaf tissues were assessed [36]: a 20 mg aliquot of frozen tissue powder was suspended in 2 mL of reaction mixture containing 0.1% (*w*/*v*) TCA, 10 mM phosphate buffer (PBS), and 1 M potassium iodide (KI) in a 1:1:2 ratio. Samples were sonicated for 10 min at 4 °C (Transsonic T460, Elma Schmidbauer GmbH, Singen, Germany) and then centrifuged (15 min, 12,000× *g*, 4 °C). The supernatant was incubated at room temperature for 20 min, and absorbance was recorded at 390 nm. H_2_O_2_ concentrations were determined using a standard curve prepared with known H_2_O_2_ concentrations (0–0.7 mM).

#### 2.4.5. Quantification of Ascorbic Acid

Ascorbic acid content in leaves was determined according to the method of Jagota & Dani [37] adapted for 96-well plate measurements: 50 mg of powdered tissue were extracted in 500 µL of 10% (*w*/*v*) TCA, vortexed, and incubated on ice for 5 min. After centrifugation (5 min, 12,000× *g*, 4 °C), 40 µL of the supernatant was diluted 1:5 in Milli-Q water, 20 µL of 0.2 M Folin–Ciocalteu reagent was added, and the mixture was allowed to react for 10 min. Absorbance was measured at 750 nm. Ascorbic acid concentration was calculated using a standard curve (0–70 µg/mL).

#### 2.4.6. Extraction in Methanol

Antioxidant activity (DPPH, ABTS) and total phenolic content (TPC) were assessed using methanolic plant extracts [33]. Briefly, 100 mg of frozen, powdered tissue was extracted with 1 mL of 75% (*v*/*v*) methanol via 15 min sonication at 35 kHz (Transsonic T460, Elma Schmidbauer GmbH, Germany). After centrifugation (10 min, 4 °C, 10,000× *g*), the supernatant was collected. The pellet was suspended in 1 mL of methanol for another extraction step performed as described above, and both extracts were pooled (total volume: 2 mL) and stored at −80 °C for analysis.

#### 2.4.7. Antioxidant Activity: DPPH Assay

For the DPPH (2,2-diphenyl-1-picrylhydrazyl) radical scavenging activity [33], 50 µL of extract was added to 1.95 mL of 0.06 mM DPPH in methanol and incubated for 30 min at room temperature. The decrease in absorbance at 520 nm indicated antioxidant activity, and the inhibition percentage was calculated by comparing the absorbance of DPPH solution with and without the plant extract, as described by Marmiroli et al. [33].

#### 2.4.8. Antioxidant Activity: ABTS Assay

To have another indication of the antioxidant activity of the plant extract, the ABTS (2,2′-azino-bis(3-ethylbenzotiazoline-6-sulfonic acid)) assay was performed following Mingle & Newsome [38], with minor modifications. The ABTS•^+^ radical was generated by reacting 0.7 mM ABTS with 2.45 mM potassium persulfate in PBS for 30 min in the dark. Plant extract (50 µL) was mixed with 150 µL of ABTS solution (adjusted to an initial absorbance of 0.7 at 734 nm) and incubated for 5-, 15-, and 30-min. Absorbance at 734 nm was measured, and inhibition percentage was calculated as for DPPH.

#### 2.4.9. Total Phenolic Content (TPC)

TPC was determined using the Folin–Ciocalteu method [33], with modifications to allow 96-well plate reading. Briefly, 10 µL of extract was incubated with 75 µL of diluted Folin–Ciocalteu reagent (1:10 in ddH_2_O) for 5 min, followed by the addition of 75 µL of 60 g/L sodium carbonate. After 90 min in the dark at room temperature, absorbance was measured at 750 nm. Results are expressed as gallic acid equivalents (GAEs) using a standard curve (0–250 µg/mL).

#### 2.4.10. Protein Extraction and Quantification

For enzyme extraction, frozen leaves (1.0 g) and roots (0.5 g) were ground in liquid nitrogen and homogenized in 5 mL and 2.5 mL of extraction buffer, respectively, containing 50 mM Tris-HCl (pH 7.8), 0.2 mM EDTA, 0.2% (*v*/*v*) Triton X-100, 1 mM PMSF, and 2 mM DTT. Homogenates were centrifuged at 12,000× *g* for 30 min at 4 °C, and the resulting supernatants were collected [36] and stored at −80 °C for total soluble protein quantification and superoxide dismutase (SOD), catalase (CAT), peroxidase (POD), and ascorbate peroxidase (APX) activity.

Total soluble protein content was quantified using the Bradford assay. The Coomassie Brilliant Blue G-250 stock dye (Bio-Rad Laboratories, Hercules, CA, USA) was diluted 1:4 with distilled water, and 40 µL of each extract was added to 2 mL of the diluted dye solution, vortexed for 10 s, and incubated for 5 min at room temperature. Absorbance was measured at 470 and 595 nm to account for spectral contributions from the dye’s different ionic forms (red, green, and blue, with absorption maxima at 470, 650, and 590 nm, respectively). Protein concentrations were calculated using a Bovine Serum Albumin (BSA) standard curve (0–1000 µg/mL) and corrected using the equation reported by Ernst & Zor [39].

#### 2.4.11. Determination of SOD Activity

SOD activity was measured by adding 100 µL of extract to 1.4 mL of reaction buffer containing 50 mM of phosphate buffer (pH 7.8), 13 mM of L-methionine, 75 µM of NBT, 0.1 mM of EDTA, and 3.3 µM of riboflavin, added last to initiate the reaction [40,41]. The mixture was exposed to fluorescent white light for 15 min to induce NBT photoreduction. A non-irradiated blank and an irradiated control without extract were included. Absorbance was read at 560 nm, and SOD activity was expressed as the percentage inhibition of NBT reduction.

#### 2.4.12. Determination of POD, CAT, and APX Activities

The activities of POD, CAT, and APX were assessed following the methods described by Shakir et al. [42]. Enzymatic activities were calculated using molar extinction coefficients of 26.6 mM/cm for POD, 39.4 mM/cm for CAT, and 2.8 mM/cm for APX. For each assay, 50 µL of enzyme extract was added to a reaction mixture with a final volume of 1.5 mL. For POD, the reaction mixture consisted of 1.35 mL of 25 mM potassium phosphate buffer (pH 7.0) containing 2 mM EDTA, 50 µL of 300 mM H_2_O_2_, and 50 µL of 1.5% (*v*/*v*) guaiacol. The increase in absorbance at 470 nm was recorded at 1, 2, and 3 min. For CAT, the mixture included 1.4 mL of 50 mM phosphate buffer (pH 7.0) with 2 mM EDTA and 50 µL of 300 mM H_2_O_2_. Absorbance at 240 nm was measured over a 30-s interval. For APX, the reaction contained 1.35 mL of 25 mM potassium phosphate buffer (pH 7.0) with 2 mM EDTA, 50 µL of 300 mM H_2_O_2_, and 50 µL of 7.5 mM ascorbate. The decrease in absorbance at 290 nm was monitored over 1 min.

#### 2.4.13. Determination of Total Soluble Sugars

The total soluble sugar content in root and leaf samples was determined following the procedure described by Shakir et al. [42], with minor modifications. Briefly, 50 mg of frozen tissue powder was homogenized in 1.5 mL of 90% ethanol and incubated for 1 h at 65 °C. The extract was then diluted to a final volume of 12.5 mL with 90% ethanol. A colorimetric reagent was prepared by mixing 5% phenol with concentrated sulfuric acid in a 1:5 ratio. Then, 250 µL of the diluted extract was combined with 1.5 mL of the reagent mixture. Absorbance was measured at 485 nm, and the total soluble sugar concentration was determined by interpolation from a glucose standard curve (20–100 µg/mL) and expressed as µg/mL.

### 2.5. Statistical Analysis

Normality and homoscedasticity of the data sets were checked with Shapiro–Wilk’s and Levene’s tests, respectively, utilizing SPSS 27 (https://www.ibm.com/it-it/products/spss-statistics, accessed on 20 November 2025).

Statistical significance among treatments was evaluated using one-way ANOVA followed by Tukey’s HSD post hoc test using Past 4.03 (©Copyright Hammer 1999–2020). Multivariate analysis, including principal component analysis (PCA), was performed using R software version 5.1 (R Core Team, 2024). The correlation matrix was created using Pearson’s method, and the relative *p*-values considered significant (*p* < 0.05) were the same as for Tukey’s test. The correlation map and the relative chord diagram were both produced using R software.

## 3. Results and Discussion

### 3.1. Influence of Biochar–PGPM Combination on Soil Microbial Activity

**Figure 1:**
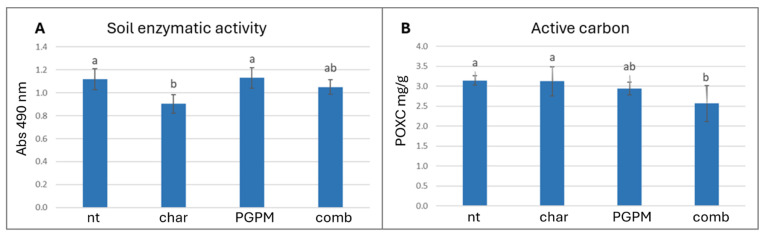
Effects of biochar (char), plant growth-promoting microorganisms (PGPMs), and their combination (comb) on (**A**) soil enzymatic activity (measured by FDA assay) and (**B**) oxidizable carbon (POXC). Different letters indicate significant differences among treatments according to Tukey’s HSD post hoc test (*p* < 0.05).

The determination of soil pH and electrical conductivity showed no significant differences among treatments, indicating that the application of both biochar and PGPMs did not perturb the overall soil physicochemical characteristics (Table 1). In contrast, the analysis of key soil health and microbial activity parameters revealed distinct patterns influenced by the differential application of PGPMs and biochar. The total hydrolytic activity of enzymes such as esterase, lipase, and protease measured by the FDA assay was reduced by about 19% in soil treated with biochar alone compared with the untreated control (Figure 1A), while total respiration (CO_2_ release) remained unaffected (Table 1). Conversely, the combination with PGPMs restored enzymatic activity to levels comparable to the control, counteracting the inhibitory effect induced by biochar. This behavior may be linked to its physicochemical properties, including its alkaline pH (9.6) and relatively high copper and nickel contents (1289 mg/kg and 119 mg/kg, respectively), which may suppress microbial activity through pH imbalance [43]. Other hypotheses are that the presence of biochar could also cause the adsorption of bioavailable carbon or could interfere with enzyme activity determination assays [44]. The combination of PGPMs with biochar not only alleviated this inhibition but also increased the consumption of oxidizable carbon (POXC) by about 18% compared to the control (Figure 1B).

In this context, biochar may act as a microbial carrier, offering porous niches that promote colonization and protection of inoculated strains, which in turn mineralize labile C fractions adsorbed to the matrix. Previous studies support this interpretation, showing that biochar combined with microbial inoculants or organic amendments enhances soil enzymatic activity [45] and promotes the stability and colonization of *Pseudomonas* spp. [46]. Interestingly, soil respiration did not differ significantly among treatments despite clear variations in enzymatic activity and labile C consumption. This apparent decoupling may reflect a microbial shift toward growth and biomass synthesis rather than CO_2_ mineralization, or the partial adsorption of CO_2_ by biochar [47]. Comparable findings were reported by Qiu et al. [48], who observed reductions in active C pools and enzymatic changes without corresponding increases in CO_2_ release. The results indicate that biochar alone reduces microbial metabolic activity, whereas inoculated microorganisms restore enzymatic activity and intensify oxidizable C consumption [49]. In a closed greenhouse pot system, the reduction in POXC does not necessarily signal soil depletion but rather reflects intensive use of available carbon by the microbial community, with potential benefits for nutrient bioavailability. The application of these amendments under field conditions could offer valuable insights into the processes governing POXC reduction, considering the role of carbon balance modulated by external environmental inputs [50].

### 3.2. Yield of Roots and Above-Ground Parts

The assessment of plant yield indicated that the applied treatments had no significant effect on plant biomass, either on shoots or roots (Table 2), despite the distinct patterns in soil carbon utilization and enzymatic activity observed under biochar and PGPM treatments. Previous studies have demonstrated that the combined application of biochar and PGPM can enhance nutrient cycling by providing suitable habitats for beneficial microorganisms and improving plant stress resilience, but such effects do not necessarily result in greater biomass accumulation [51,52]. Furthermore, biomass and fresh/dry weight responses may require longer-term observations to become evident, particularly if biochar primarily enhances physiological mechanisms related to stress tolerance or nutrient efficiency during the early stages of growth [53].

### 3.3. Roots to Shoots Redox Responses and Oxidative Stress Indicators

**Figure 2:**
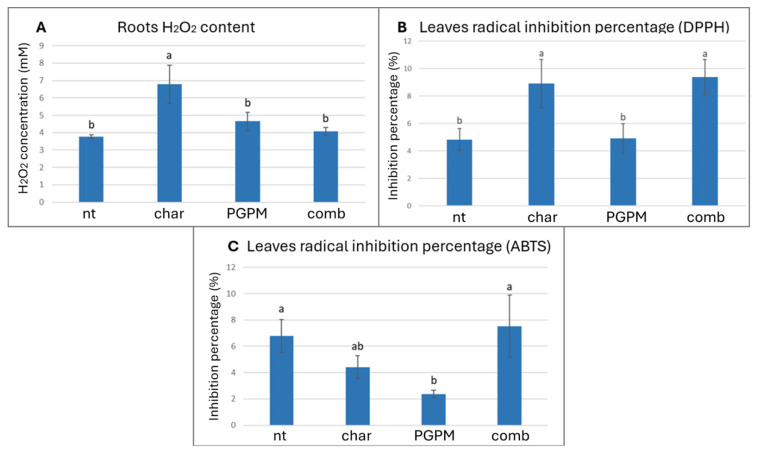
Effects of biochar (char), plant growth-promoting microorganisms (PGPMs), and their combination (comb) on (**A**) roots’ H_2_O_2_ content, (**B**) leaves’ radical inhibition percentage measured by DPPH assay, and (**C**) leaves’ radical inhibition percentage measured by ABTS assay. Different letters indicate significant differences among treatments according to Tukey’s HSD post hoc test (*p* < 0.05).

Analyses of root tissues revealed an increase in H_2_O_2_ content in plants exposed to biochar alone (Figure 2A), with levels rising by 55.4% compared to the control, while H_2_O_2_ accumulation in leaves remained unchanged (Table 3). Interestingly, this localized rise in root H_2_O_2_ did not coincide with any significant increase in lipid peroxidation in either roots or leaves, or alteration in leaves’ cellular respiration (Table 3). It is well known that moderate accumulation of H_2_O_2_ appears to function as a key redox signal able to mediate plant stress acclimation: its controlled increase activates ROS-dependent signaling cascades and redox-sensitive proteins, leading to transcriptional and translational adjustments consistent with enhanced tolerance [54]. The lack of oxidative damage in plants treated with biochar suggests that the detected H_2_O_2_ increase in roots reflects an adaptive rather than damaging response. This mechanism is supported by previous evidence showing that moderate H_2_O_2_ levels modulate hormonal pathways, particularly abscisic acid signaling, and promote antioxidant reinforcement [55,56]. When biochar was combined with PGPMs, H_2_O_2_ accumulation in roots was attenuated, suggesting that microbial inoculation contributed to the mitigation of biochar-induced oxidative response. This effect reflects the capacity of several PGPMs, including *Pseudomonas* and *Azotobacter*, to alleviate redox imbalance through the secretion of antioxidant metabolites or by priming host defense pathways [57,58,59]. The combined biochar–PGPM treatment thus appears to promote a more balanced redox homeostasis in roots, reducing stress intensity while maintaining signaling competence. At the foliar level, antioxidant assays based on radical inhibition revealed distinct patterns: the DPPH assay showed higher inhibition percentages in leaves of plants treated with biochar, either alone or combined with PGPMs (8.9% and 9.4%, respectively), compared to both the PGPM-only and control treatments (4.9% and 4.8%, respectively; Figure 2B). Conversely, the ABTS assay revealed no difference under biochar alone compared to the control, but a significant reduction in radical inhibition under PGPMs alone, dropping from 6.8% in the control to 2.4% in the treated plants (Figure 2C). This divergence between DPPH and ABTS responses may arise from their different chemical sensitivities [60]: DPPH primarily detects lipophilic antioxidants and certain phenolic classes, while ABTS also responds to hydrophilic antioxidants [61,62]. The selective increase in DPPH inhibition suggests that biochar, alone or in combination with PGPMs, stimulated the synthesis and accumulation of lipophilic antioxidant compounds without substantially affecting hydrophilic antioxidant pools. This result may indicate a possible priming mechanism in which biochar-induced H_2_O_2_ accumulation in roots acts as a mobile redox signal, activating antioxidant metabolism in leaves through hormone-mediated signaling networks involved in abiotic stress tolerance [63,64]. This mechanism would enhance plant resilience against oxidative stress by promoting antioxidant accumulation prior to the induction of oxidative damage [65].

### 3.4. Antioxidant Modulation in Roots and Leaves

**Figure 3:**
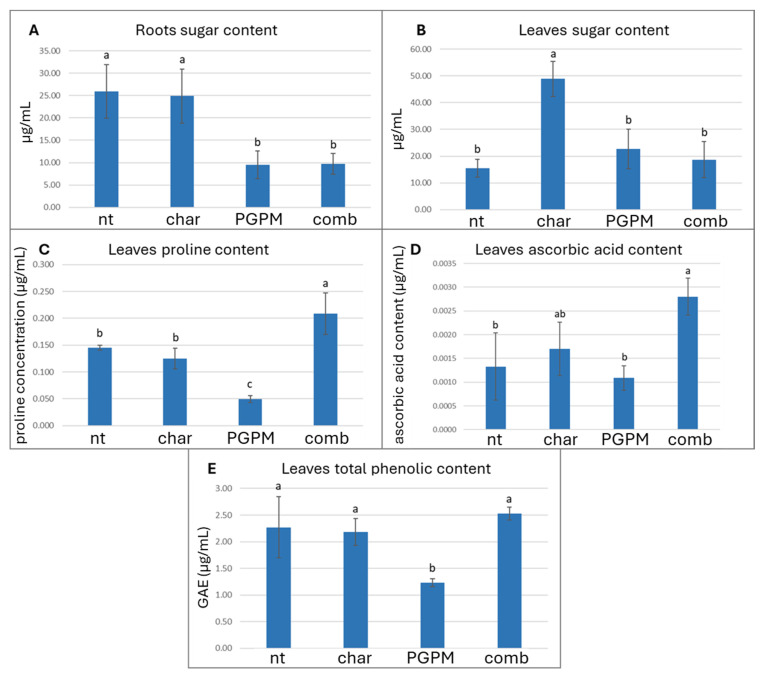
Effects of biochar (char), plant growth-promoting microorganisms (PGPMs), and their combination (comb) on (**A**) roots’ total soluble sugar content, (**B**) leaves’ total soluble sugar content, (**C**) leaves proline content, (**D**) leaves’ ascorbic acid content, and (**E**) leaves’ total phenolic content (TPC). Different letters indicate significant differences among treatments according to Tukey’s HSD post hoc test (*p* < 0.05).

The assessment of total soluble sugar content in roots and leaves serves as a useful indicator of the plant antioxidant and metabolic response, reflecting their function as osmoprotectants and modulators of ROS dynamics via sugar-derived signaling pathways, particularly in systems affected by PGPM and biochar interactions. At the root level, the inoculation with PGPMs, either alone or in combination with biochar, resulted in a marked reduction (approximately 37%) in soluble sugar concentrations compared with the uninoculated control (Figure 3A). This decrease may reflect enhanced microbial metabolic activity and carbon demand in the rhizosphere, with plant-derived carbohydrates being mobilized to sustain microbial proliferation and root colonization. This interpretation aligns with the observed decline in soil oxidizable carbon (POXC), indicating intensified use of labile carbon fractions by the plant–microbe system. The concurrent depletion of both root sugars and POXC supports the notion that PGPMs promote a dynamic carbon exchange between plant tissues and soil microbial communities, thereby accelerating carbon turnover and improving resource efficiency in the rhizosphere. Comparable responses have been documented in previous studies, where PGPM inoculation stimulated the mobilization of stored carbohydrates in roots, leading to a reduction in sugar reserves that facilitated symbiotic interactions and rhizodeposition processes [58,66]. Beyond this metabolic role, soluble sugars also function as pivotal signaling molecules in root development, energy allocation, and stress-related pathways [67]. Therefore, the observed decline in root sugars may not only represent enhanced microbial consumption but also a PGPM-induced metabolic reprogramming aimed at optimizing carbon fluxes and enhancing the plant’s physiological resilience [68]. Indeed, several PGPM strains have been shown to modulate carbon metabolism by adjusting sugar partitioning between shoots and roots, enhancing carbon allocation to metabolically active tissues, and improving carbon use efficiency [69]. The pattern observed in leaves displayed an opposite trend: under biochar alone, soluble sugar concentrations increased significantly by approximately 31.7% compared with the control (Figure 3B), suggesting a possible shift in carbon partitioning between below- and above-ground organs. This behavior may reflect reduced root carbon demand and enhanced allocation of photoassimilates to the shoots, potentially driven by improved soil physical conditions and nutrient availability associated with biochar amendment. Alternatively, the accumulation of soluble sugars in leaves may represent an adaptive strategy to enhance osmotic balance and to provide precursors for secondary metabolite biosynthesis, both of which contribute to stress tolerance [68,69]. Notably, the increase in leaf sugar content coincided with higher root H_2_O_2_ levels, suggesting a potential signaling link between oxidative stress and carbohydrate metabolism. Exogenous or endogenous H_2_O_2_ generated in roots is known to act as a systemic signal that triggers sugar accumulation in leaves, thereby priming plants to counteract abiotic stress conditions through the reinforcement of antioxidant and protective metabolic pathways [64]. This increase in root H_2_O_2_ content observed under biochar alone did not translate into significant changes in the activity of key antioxidant enzymes (SOD, POD, CAT, and APX) in either roots or leaves, nor did it interfere with photosynthetic activity (Table 4). Similarly, total protein content remained statistically unchanged across treatments in both organs (Table 4), indicating that the oxidative signal induced by biochar was not associated with generalized protein degradation or stress-induced metabolic suppression. In contrast, the non-enzymatic antioxidant profile assessed through proline (Figure 3C), ascorbic acid (Figure 3D), and total phenolic content (Figure 3E), revealed a distinct response. Biochar alone had a limited influence on these metabolites, while PGPM inoculation alone resulted in a significant reduction in all three parameters (66.0%, 18.0%, 45.7%, respectively), indicating a downregulation or redistribution of antioxidant and osmolyte metabolism. Interestingly, when PGPMs were combined with biochar, however, metabolite levels were restored to values comparable to, or even exceeding, those of the control, suggesting that the presence of biochar mitigated the reduction induced by PGPMs alone. This pattern supports the hypothesis of a priming mechanism, wherein the moderate H_2_O_2_ accumulation observed in roots exposed to biochar may function as a systemic signal that activates antioxidant defenses in the leaves through hormonal and redox-mediated signaling pathways associated with abiotic stress tolerance [63,64] prior to the induction of enzymatic defenses or oxidative damage [65]. Furthermore, different species of PGPMs (like *Pseudomonas* spp.) are known to trigger secondary metabolism, stimulating phenolic and flavonoid synthesis through defense pathway activation [57,70]. The enhanced antioxidant response observed under the combined biochar–PGPM treatment, in contrast to the suppression seen with PGPMs alone and the limited effects of biochar alone, suggests a synergistic interaction between the two components: biochar may act as a physical and chemical reservoir for microbial metabolites or signaling molecules, prolonging their bioavailability in the rhizosphere and amplifying their systemic effects on plant metabolism, optimizing redox balance and plant capacity for metabolic adjustment under stress-related conditions.

### 3.5. Integrated Multivariate Insights into Plant–Soil System: A Decryption Key

**Figure 4:**
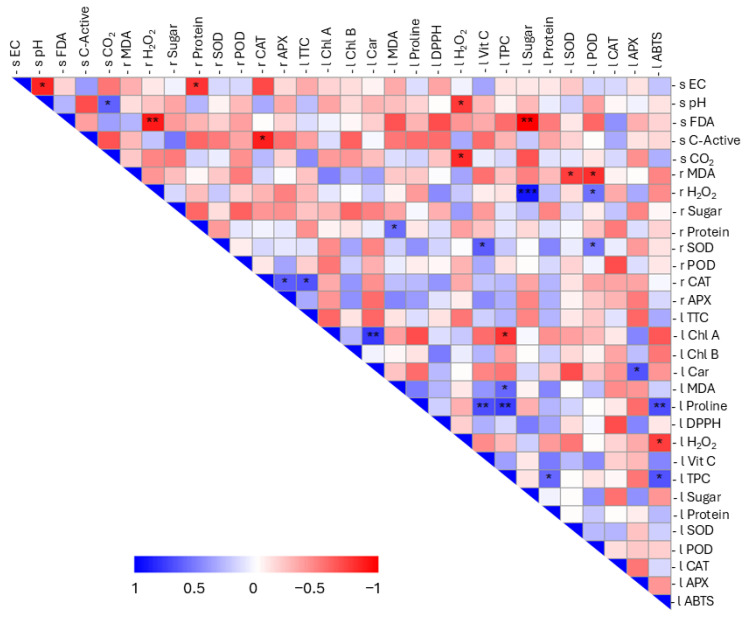
Pearson correlation matrix between the analysed parameters. Positive correlations are shown in blue and negative correlations in red. The colour intensity is proportional to the correlation value, as shown by the scale below (from 1 to −1). Significant correlations are highlighted with asterisks: *p*-value <0.05 (*); *p*-value <0.01 (**); and *p*-value <0.001 (***). The letters s, l, and r were used to represent soil, leaves, and roots, respectively. To distinguish between the various substrates of the matrix under consideration, the parameters were assigned a letter: s for soil, r for roots, and l for leaves.

Multivariate analysis provided an integrated view of the plant–soil system, revealing consistent patterns across scales. Pearson correlation was applied to assess the correlation among different physiological and biochemical attributes, which were schematized in a correlation matrix and a chord diagram (Figure 4 and Figure S2).

With regard to soil variables, negative correlations were identified between EC, pH, and protein content in roots (r Protein). In addition, pH was positively correlated with soil CO_2_ content and negatively correlated with H_2_O_2_ content in leaf samples (l H_2_O_2_). Enzymatic activity (s FDA) showed a strong negative correlation with root H_2_O_2_ (r H_2_O_2_) and leaf sugar content (l Sugar), with *p* values of 0.009 and 0.001, respectively. A negative correlation was also observed between active C and root catalase (r CAT). Soil respiration (s CO_2_) was negatively correlated with l H_2_O_2_. For plant parameters, a negative correlation was found between lipid peroxidation in roots (r MDA) and certain antioxidant enzymes in leaves (l SOD; l POD). Conversely, leaf lipid peroxidation (l MDA) was positively correlated with total phenolic content (l TPC) and with root protein content (r Protein). Root hydrogen peroxide content exhibited a positive correlation with l POD and was also closely correlated with leaf sugar levels (*p* = 0.00003). Positive correlations among enzymatic parameters were evident, including r SOD with l POD and l vitamin C; r CAT and r APX with cell viability (l TTC); and, in leaves, l APX with one of the photosynthetic pigments (l Car). Regarding pigments, a robust positive correlation (*p* = 0.002) was identified between chlorophyll a (l Chl A) and carotenoid content (l Car), accompanied by a negative correlation with l TPC. Proline levels (l Proline) showed a strong positive correlation with biochemical indicators of antioxidant status, namely l ABTS, l TPC, and l Vit C, with *p* values of 0.009, 0.003, and 0.008, respectively. The correlation between leaf sugar content and root H_2_O_2_ suggests that increased hydrogen peroxide in roots is associated with enhanced primary metabolic activity, leading to elevated sugar accumulation in leaves, a metabolic interaction consistent with previous reports [64]. A contrasting relationship between these two parameters (root hydrogen peroxide and leaf sugar content) emerged in the context of soil enzymatic activity, a pattern not yet explored in depth in the literature and potentially offering a starting point for future mechanistic studies. Proline content in leaves was positively correlated with ABTS reduction, total phenolic content, and vitamin C content, indicating a synergistic antioxidant response to oxidative stress. In tomato plants, it is well documented that proline content correlates positively with vitamin C, phenolic content, and antioxidant activity measured by the ABTS assay [71]. Moreover, a positive correlation between Chl A and carotenoids was also observed, highlighting the co-regulation of chlorophyll and carotenoid biosynthesis during plastid development and pigment accumulation, as well as the potential accumulation of photosynthates in the fruit [72].

**Figure 5:**
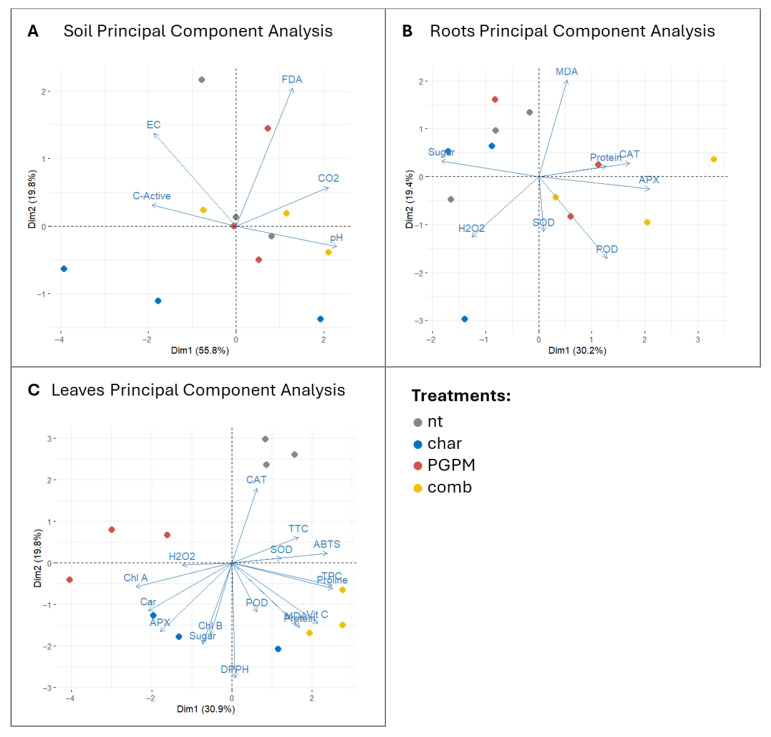
Principal component analysis (PCA) of soil (**A**), root (**B**), and leaf (**C**) parameters under different treatments: untreated control (nt), biochar (char), plant growth-promoting microorganisms (PGPMs), and their combination (comb). Vectors indicate the contribution of individual variables to the variance, while points represent the distribution of treatments across principal components.

PCA performed on the different datasets clearly separated the four experimental conditions, identifying the microbial inoculum and the combined biochar strategy as the main drivers of system perturbation. In the soil dataset (Figure 5A), the microbial component, explaining 55.8% of the total variance, was associated with changes in soil-level activity, whereas the second component, related to biochar application and accounting for 19.8% of the total variance, aligned with previous findings attributing such effects to biochar’s adsorptive properties or the release of potentially toxic compounds [43,44]. When biochar was combined with PGPMs, microorganisms appeared to exploit biochar surfaces as protective niches, ultimately contributing to the restoration of soil metabolic dynamics and highlighting the effectiveness of this combined strategy [45]. This evidence supports the concept of biochar functioning as an ecological carrier that enhances microbial resilience when co-applied with beneficial microorganisms [73]. Root responses provided further insight (Figure 5B), with the microbial factor (alone or in combination) emerging as a major source of variation (30.2% of the total variance), while biochar alone was projected along axes dominated by H_2_O_2_, confirming a localized oxidative stress response. In contrast, inoculated treatments, particularly the biochar–PGPM combination, were associated with metabolic and protein-related parameters, indicating a broader metabolic adjustment. This outcome can be explained by the release of phytohormones and metabolites by PGPMs such as *Pseudomonas fluorescens* and *Azotobacter chroococcum*, which contribute to plant carbon demand and redox balance regulation [58,74]. The reduction in root sugars under PGPM treatments, also captured by PCA, may therefore reflect not only microbial consumption but also plant-driven reallocation of carbon towards defense and shoot growth, in line with the signaling role of sugars [68,69]. In leaves (Figure 5C), all treatments were clearly separated, predominantly along gradients defined by antioxidant traits, whereas single-factor treatments were less discriminant, with the first two components together explaining only 51.7% of the total variance. This pattern suggests that the combined biochar–PGPM treatment elicits a systemic antioxidant response, likely aimed at counteracting potential oxidative stress through enhanced ROS scavenging [75].

These findings provide a statistical interpretation of the complex system under investigation and indicate that the biochar–PGPM interaction may trigger systemic priming without measurable oxidative damage, whereby root-derived ROS and hormonal signals induce the accumulation of defensive metabolites in shoots, preparing plants for environmental stress while preserving cellular homeostasis [63,65]. The selective increase in antioxidant activity detected by the DPPH assay, which reflects lipophilic antioxidant pools, supports the activation of secondary metabolic pathways associated with long-term adaptation [61,62]. The absence of these effects under PGPMs alone, together with the limited responses observed under biochar alone, highlights the synergistic nature of the combined treatment in both mitigating root oxidative stress and activating systemic defense responses. Taken together, the three PCA analyses converge on a coherent interpretation: biochar alone introduces potentially detrimental perturbations; PGPMs alone partially improve root status without inducing strong systemic effects; whereas their combination converts biochar-induced stress into a beneficial signal that primes systemic defense. This underscores the importance of evaluating the integrated plant–soil level, while accounting for dynamic environmental interactions [76].

Several biochemical variables showed clear trends; however, a number of parameters did not reach statistical significance (*p* > 0.05), reflecting the inherent variability of pot experiments and the absence of imposed abiotic or biotic stress. In addition, the experiment lasted only three months and did not include measurements of fruit yield or quality, thereby constraining the assessment of physiological and biochemical responses to the vegetative growth stage. These limitations restrict the extrapolation of the findings to broader field conditions and longer cropping cycles. Future work should therefore incorporate defined stress treatments, extended field trials, and metabolomic profiling for higher biochemical resolution, together with yield-level assessments.

## 4. Conclusions

From an applied perspective, the co-application of biochar and PGPMs emerges as a promising and sustainable strategy that may potentially prime plants toward enhanced defensive capacity, particularly under the increasing frequency of environmental stressors associated with climate change. As global agriculture faces intensified drought, heat, and nutrient imbalances, optimizing beneficial interactions within the plant–soil system will be crucial to improve resource-use efficiency, reduce dependence on chemical inputs, and sustain productivity in degraded or marginal soils. Future field-scale investigations should therefore aim to verify whether these greenhouse results translate into yield benefits and improved stress tolerance under realistic climatic fluctuations and across diverse crop systems, although current evidence strongly supports their integrated use as a tool for future agriculture.

## Figures and Tables

**Table 1:** Mean values (up) and standard deviation (down) obtained for each treatment in the corresponding analyses in soil, along with the *p*-values from the one-way ANOVA, for cases where the differences were not significant.

Soil Samples	Untreated (nt)	Biochar (char)	PGPM	Biochar + PGPM (comb)	*p*-Value
Electrical Conductivity (µS/cm^2^)	**2817.2** ±1405.5	**2647.2** ±1365.9	**2166.9** ±612.7	**2607.5** ±1125.2	**0.91**
pH	**7.53** ±0.20	**7.35** ±0.74	**7.51** ±0.12	**7.51** ±0.34	**0.95**
Soil respiration (mg CO_2_)	**8.91** ±0.47	**7.61** ±1.78	**8.53** ±1.14	**9.30** ±0.13	**0.33**

Bold indicates EM values, normal style indicates SE.

**Table 2:** Mean values (up) and standard deviation (down) obtained for each treatment of roots fresh weight and shoots fresh and dry weight, along with the *p*-values from the one-way ANOVA.

Weight	Untreated (nt)	Biochar (char)	PGPM	Biochar + PGPM (comb)	*p*-Value
Roots fresh weight (g)	**12.5** ±2.78	**8.0** ±0.56	**11.0** ±1.41	**6.0** ±0.74	**0.45**
Shoots fresh weight leaves and stems (g)	**275.0** ±35.36	**212.5** ±95.46	**232.5** ±10.61	**255.0** ±24.04	**0.68**
Shoots dry weight leaves and stems (g)	**26.04** ±8.78	**23.05** ±0.47	**28.50** ±5.71	**21.71** ±1.28	**0.61**

Bold indicates EM values, normal style indicates SE.

**Table 3:** Mean values (up) and standard deviation (down) obtained for each treatment in the corresponding analyses in leaves and roots, along with the *p*-values from the one-way ANOVA, for cases where the differences were not significant.

Oxidative Stress Parameters	Untreated (nt)	Biochar (char)	PGPM	Biochar + PGPM (comb)	*p*-Value
Roots lipidic peroxidation−MDA assay (ng/mL)	**1.15** ±0.13	**1.15** ±0.27	**1.25** ±0.25	**1.17** ±0.15	**0.91**
Leaves lipidic peroxidation − MDA assay (ng/mL)	**10.1** ±2.2	**10.9** ±0.6	**10.2** ±0.9	**12.5** ±1.3	**0.16**
Leaves H_2_O_2_ content (µM)	**3.95** ±0.33	**4.55** ±0.99	**4.13** ±0.19	**3.45** ±0.62	**0.40**
Leaves cellular respiration − TTC assay *(Abs λ =* 530 nm)	**0.26** ±0.036	**0.23** ±0.028	**0.22** ±0.052	**0.27** ±0.086	**0.65**

Bold indicates EM values, normal style indicates SE.

**Table 4:** Mean values (up) and standard deviation (down) for each treatment in the corresponding analyses in leaves and roots, along with the *p*-values from the one-way ANOVA, for cases where the differences were not significant.

Antioxidants Parameters	Untreated (nt)	Biochar (char)	PGPM	Biochar + PGPM (comb)	*p*-Value
Chlorophyll A content (mg/g FW)	**0.50** ±0.004	**0.53** ±0.029	**0.55** ±0.030	**0.51** ±0.024	**0.11**
Chlorophyll B content (mg/g FW)	**0.54** ±0.091	**0.69** ±0.068	**0.72** ±0.052	**0.75** ±0.104	**0.06**
Carotenoids content (mg/g FW)	**0.164** ±0.009	**0.174** ±0.004	**0.178** ±0.009	**0.169** ±0.008	**0.21**
Leaves total protein content (BSA µg/mL)	**392.03** ±28.74	**438.13** ±83.61	**387.57** ±38.26	**470.62** ±33.08	**0.22**
Leaves SOD activity (I%)	**54.77** ±13.79	**52.29** ±4.44	**50.56** ±3.88	**55.89** ±5.22	**0.84**
Leaves POD activity (U/mL)	**0.096** ±0.018	**0.127** ±0.033	**0.109** ±0.036	**0.115** ±0.032	**0.68**
Leaves CAT activity (U/mL)	**0.205** ±0.085	**0.086** ±0.047	**0.108** ±0.013	**0.103** ±0.029	**0.36**
Leaves APX activity (U/mL)	**0.272** ±0.071	**0.539** ±0.111	**0.545** ±0.250	**0.401** ±0.128	**0.18**
Roots total protein content (BSA µg/mL)	**223.11** ±50.95	**225.45** ±18.34	**247.83** ±23.98	**251.82** ±9.58	**0.56**
Roots SOD activity (I%)	**45.01** ±3.00	**47.70** ±2.55	**44.31** ±3.63	**48.90** ±6.18	**0.50**
Roots POD activity (U/mL)	**0.634** ±0.10	**0.661** ±0.11	**0.705** ±0.20	**0.739** ±0.08	**0.78**
Roots CAT activity (U/mL)	**0.043** ±0.012	**0.063** ±0.040	**0.048** ±0.031	**0.124** ±0.099	**0.33**
Roots APX activity (U/mL)	**0.925** ±0.080	**0.896** ±0.338	**0.921** ±0.171	**1.354** ±0.291	**0.14**

Bold indicates EM values, normal style indicates SE.

## Data Availability

All data are available upon request to the authors.

## Supplementary Materials

The following supporting information can be downloaded at: https://www.mdpi.com/article/10.3390/antiox14121482/s1, Table S1. Physico-chemical characterization of biochar utilized in the experiments. Details on the methods utilized are reported in Marmiroli et al. (2022) [77]. Figure S1. Structures (A) and EDX spectra of biochar (B). Biochar was assessed using an ESEM FEG2500 FEI Environmental Scanning Electron Microscope (FEI Europe, Eindhoven, Netherlands) with energy-dispersive X-ray spectroscopy (EDX). Methods utilized are described in Marmiroli et al. (2022) [77]. Figure S2. A chord diagram illustrating the significant interactions between the analysed parameters (*p* < 0.05). Positive correlations are indicated by blue chords, while negative correlations are indicated by red chords. Statistically significant correlations with *p* < 0.01 are highlighted with a black border, while those with *p* < 0.001 are highlighted with a thick, dotted black line along the respective chords.
