# Supplementary material for: Analyses of Antioxidative Response in Tomato (Solanum lycopersicum L.) Grown with Biochar and PGPMs
Source: Antioxidants (Basel). 2025 Dec 10;14(12):1482. doi: 10.3390/antiox14121482 (PMC12729948; doi:10.3390/antiox14121482)
Supplement: Supplementary file 1 [file antioxidants-14-01482-s001.zip › antioxidants-4016191-supplementary.pdf]

Supplementary Materials

Analysis of Antioxidative Response in Tomato (*Solanum lycopersicum* L.) Grown with Biochar and PGPM

Silvia Carlo [1], Marta Trazza [1], Luca Pagano [2] and Marta Marmiroli [1]

[1] Department of Chemistry, Life Sciences and Environmental Sustainability, University of Parma, 43124 Parma, Italy; silvia.carlo@unipr.it (S.C.); marta.trazza@unipr.it (M.T.)
[2] Consorzio Interuniversitario Nazionale per le Scienze Ambientali (CINSA), University of Parma, 43124 Parma, Italy; luca.pagano@unipr.it
* Correspondence: marta.marmiroli@unipr.it

Table S1. Physico-chemical characterization of biochar utilized in the experiments. Details on the methods utilized are reported in Marmiroli et al., 2022 [1].

Analysis	Data
pH	9.57 ± 0.02
Electric Conductivity (mS/m)	110.63 ± 9.26
Density (g/cm^3)	0.36 ± 0.017
Organic Matter (%)	87
Metal Content (mg/kg)	Cd: 0.573 ± 0.03<br>Ni: 119.70 ± 32.93<br>Cu: 1289.38 ± 165.53<br>Zn: 127.97 ± 14.25<br>Fe: 3380.42 ± 231.87

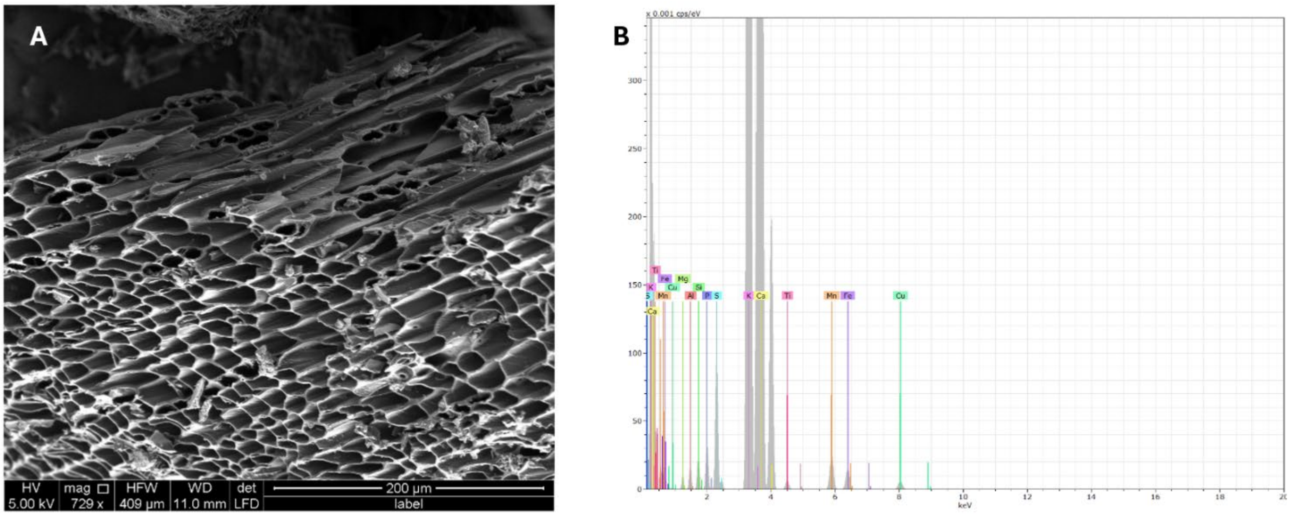

Figure S1. Structures (**A**) and EDX spectra of biochar (**B**). Biochar showed at Environmental Scanning Electron Microscope ESEM FEG2500 FEI (FEI Europe, Eindhoven, The Netherlands) with energy dispersive X-ray spectroscopy (EDX). Methods utilized are described in Marmiroli et al., 2022 [1].

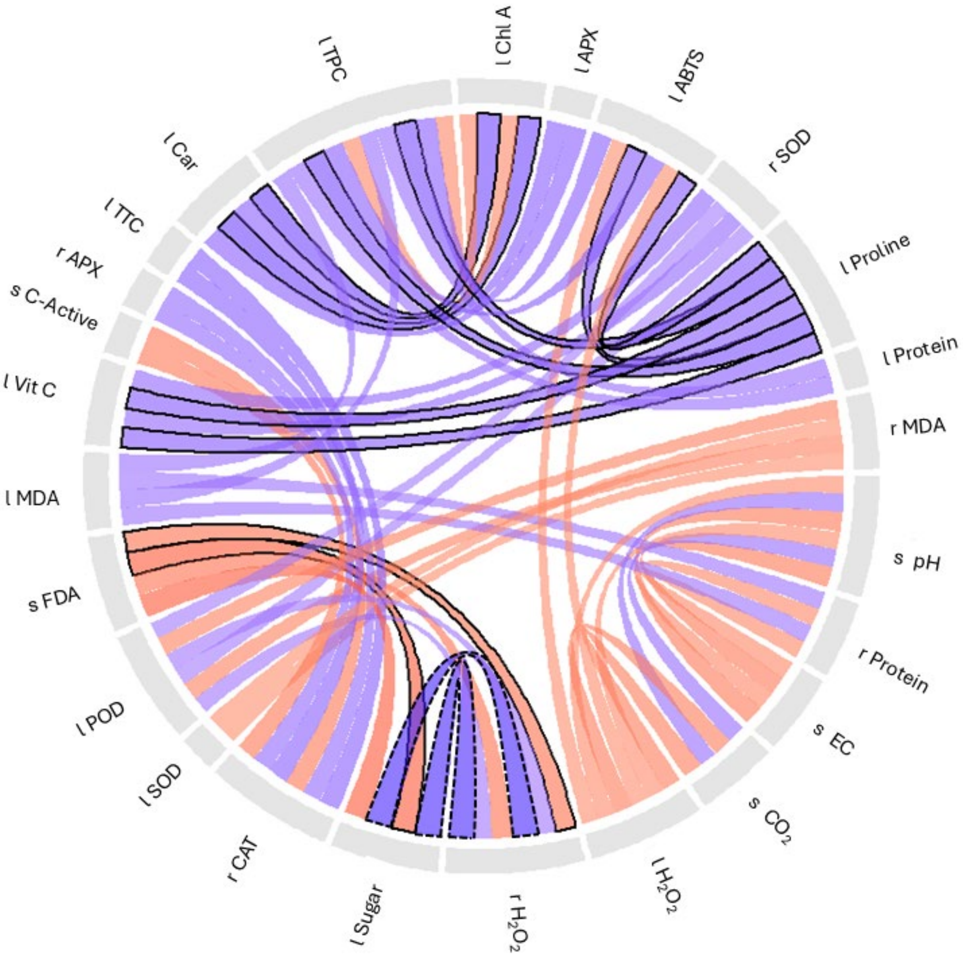

Figure S2. Chord diagram that represents the significant interactions between the analysed parameters ($p < 0.05$). Positive correlations are indicated by blue chords, while negative correlations are indicated by red chords. Statistically significant correlations with $p < 0.01$ are highlighted with a black border, while those with $p < 0.001$ are highlighted with a thick, dotted black line along the respective chords.

Reference

1. Marmiroli M.; Caldara M.; Pantalone S.; Malcevschi A.; Maestri E.; Keller A.A.; Marmiroli N. Building a risk matrix for the safety assessment of wood derived biochars. *Sci. Total Environ*. **2022**, *839*, 156265. https://doi.org/110.1016/j.scitotenv.2022.156265.
